# Fatty Infiltration of Skeletal Muscle: Mechanisms and Comparisons with Bone Marrow Adiposity

**DOI:** 10.3389/fendo.2016.00069

**Published:** 2016-06-20

**Authors:** Mark W. Hamrick, Meghan E. McGee-Lawrence, Danielle M. Frechette

**Affiliations:** ^1^Department of Cellular Biology and Anatomy, Medical College of Georgia, Augusta, GA, USA; ^2^Department of Biomedical Engineering, Stony Brook University, Stony Brook, NY, USA

**Keywords:** bone marrow adipogenesis, myosteatosis, intramyocellular lipid, exercise

## Abstract

Skeletal muscle and bone share common embryological origins from mesodermal cell populations and also display common growth trajectories early in life. Moreover, muscle and bone are both mechanoresponsive tissues, and the mass and strength of both tissues decline with age. The decline in muscle and bone strength that occurs with aging is accompanied in both cases by an accumulation of adipose tissue. In bone, adipocyte (AC) accumulation occurs in the marrow cavities of long bones and is known to increase with estrogen deficiency, mechanical unloading, and exposure to glucocorticoids. The factors leading to accumulation of intra- and intermuscular fat (myosteatosis) are less well understood, but recent evidence indicates that increases in intramuscular fat are associated with disuse, altered leptin signaling, sex steroid deficiency, and glucocorticoid treatment, factors that are also implicated in bone marrow adipogenesis. Importantly, accumulation of ACs in skeletal muscle and accumulation of intramyocellular lipid are linked to loss of muscle strength, reduced insulin sensitivity, and increased mortality among the elderly. Resistance exercise and whole body vibration can prevent fatty infiltration in skeletal muscle and also improve muscle strength. Therapeutic strategies to prevent myosteatosis may improve muscle function and reduce fall risk in the elderly, potentially impacting the incidence of bone fracture.

## Introduction

Osteoporosis affects ~10 million people in the U.S. and results in over 1.5 million bone fractures per year. Hip fractures are a major cause of morbidity and mortality among the elderly: ~40% of those suffering a hip fracture will end up in a nursing home and 20% will never walk again. In addition, the 1-year mortality of hip fractures at age 70 is ~30%. Muscle weakness and postural instability are major contributors to the incidence of falls among the elderly, and falling is the primary etiological factor in more than 75% of hip fractures (1). Loss of muscle and bone mass with age is therefore a significant public health problem, as the morbidity that accompanies fractures in the elderly is costly both in terms of financial burden and quality of life. The mechanisms underlying loss of muscle and bone strength with age are complex and multifactorial in nature, but evidence suggests that common factors regulate the integrated growth, development, and degeneration of these two tissues. For example, skeletal muscle and bone share common embryological origins from mesodermal cell populations and also display common growth trajectories early in life. Moreover, muscle and bone are both mechanoresponsive tissues, and the mass and strength of both tissues decline with age. Importantly, the decline in muscle and bone strength that occurs with aging is accompanied in both cases by an accumulation of adipose tissue. This accumulation of fat in non-adipose depots, such as bone, liver, and muscle, is now recognized as a common feature of aging (2). The processes driving the accumulation of bone marrow adipocytes (ACs) are becoming more well understood (3, 4); however, the factors leading to the accumulation of fat in skeletal muscle (myosteatosis) with age are not yet as well defined. Evidence, to date, does suggest that many of the factors that have been observed to stimulate bone marrow adipogenesis, such as estrogen deficiency, glucocorticoid treatment, and disuse atrophy, also induce myosteatosis. In this study, we review these findings to highlight potential therapeutic strategies for the prevention of age-related myosteatosis as an approach for reducing fall risk and hence the likelihood of bone fracture.

## Factors Contributing to Bone Marrow Adipogenesis

Bone cell populations are heterogeneous and include cells of both hematopoietic (e.g., megakaryocytes and osteoclasts) and mesenchymal (e.g., osteoblasts and AC) origin. Aging is accompanied by an accumulation of AC as well as increase in ACs size within the bone marrow cavity (5). Adipose tissue represents ~20% of bone marrow tissue before the third decade in life but increases to nearly 50% by the ninth decade (6). This accumulation of bone marrow fat shows a strong association with bone loss, reduced bone formation, and fracture risk (6–9). Mesenchymal progenitors (MSCs) within bone marrow can be directed toward the AC or osteoblast lineage, and conditions that favor adipogenesis such as estrogen depletion (10), disuse (11), anorexia/calorie restriction (12, 13), and exposure to microgravity (14) are also associated with reduced osteoblast differentiation.

In addition, there are a number of pharmaceutical treatments that can mediate bone marrow adipogenesis. For example, glucocorticoids and PPAR gamma agonists will stimulate adipogenesis in mesenchymal progenitors (15, 16), whereas lipid-lowering statins can inhibit adipogenic differentiation (17). Importantly, the microenvironment of the MSCs plays a key role in modulating this reciprocal switch between adipogenic or osteogenic differentiation, particularly with aging, as young MSCs transplanted into old animals or young MSCs exposed to serum of old donors will tend to differentiate down the adipogenic pathway rather than become osteogenic (18, 19). Finally, epigenetic programing also appears to play an important role in modulating bone marrow adipogenesis. For example, conditional deletion of Hdac3 in preosteoblasts increases marrow AC number and lipid storage in preosteoblasts (20). It is worth noting that marrow ACs are themselves not homogenous in their gene expression and secretory profile. For example, some marrow ACs are similar to “white” fat in being rich in saturated fatty acids, whereas other marrow ACs are more “beige-like” fat in having greater thermogenic potential (4).

## Fatty Infiltration in Skeletal Muscle: Cellular and Molecular Mechanisms

**Figure 1:**
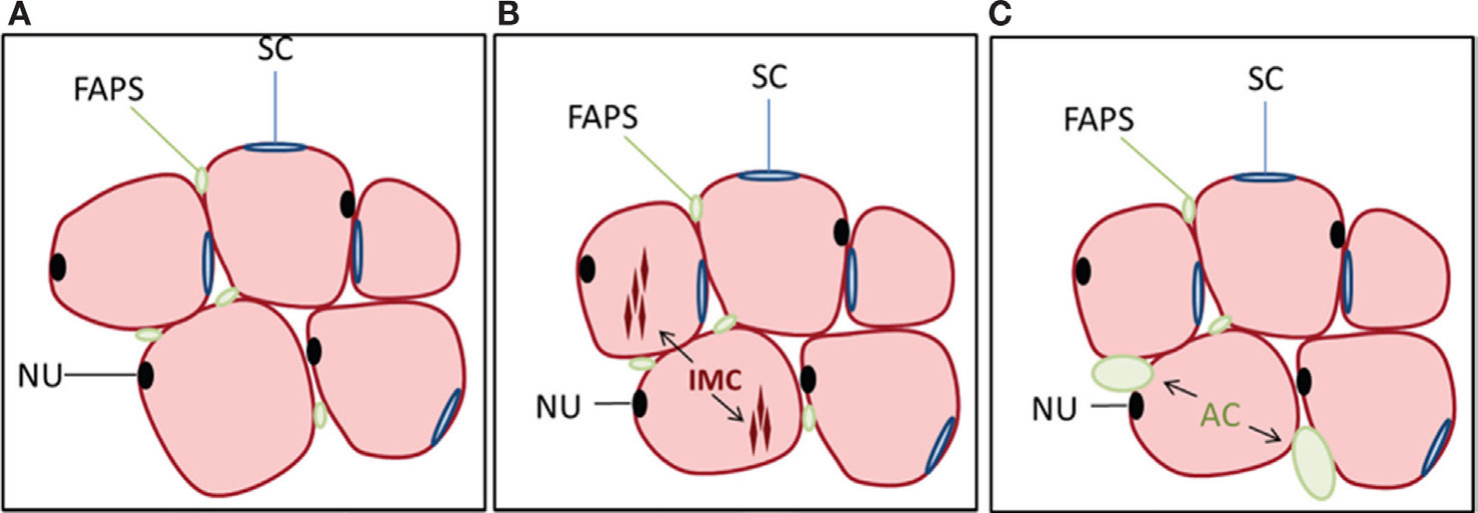
**Cell populations in muscle and their relationship to lipid accumulation**. **(A)** Myofibers (pink) are multinucleated (NU, nucleus, black) and surrounded by satellite cells (SCs, blue) as well as multipotential cells of mesenchymal origin referred to as fibro-adipogenic progenitors (FAPs, green). FAPs are distinct from satellite cells and lack Pax7 expression but are Sca-1 and PDGFRα positive. Not shown are pericytes surrounding blood vessels within muscle. **(B)** Intramyocellular (IMC) lipid can accumulate within myofibers, which is one pathway for lipid deposition within skeletal muscle. **(C)** FAPs can also differentiate to adipocytes (ACs), contributing to the accumulation of intermuscular fat, often following muscle injury.

Aging in humans is accompanied by a loss of subcutaneous fat but an accumulation of AC and lipids in non-adipose depots, such as bone marrow, liver, and skeletal muscle (2). Fatty infiltration of skeletal muscle (myosteatosis) has, in particular, been recognized as an important component of aging and frailty (21–26). Lipid accumulation in muscles of the lower limb is also associated with increased fracture risk in the elderly (27). The cellular origins of fatty accumulation in muscle arise through several different pathways (Figure 1). One direct route is *via* the accumulation of lipid within myofibers themselves, known as intramuscular fat or intramyocellular (IMC) lipid (28–30). Accumulation of IMC lipid is now known to be associated with insulin insensitivity, inflammation, and functional deficits in skeletal muscle. Accumulation of the sphingolipid ceramide appears to have a particularly detrimental effect on skeletal muscle function (30). Recent data also suggest that the lipid metabolites diacylglycerols (DAG) are responsible for mediating insulin resistance in skeletal muscle through disrupting the insulin signaling pathway (31).

Another pathway for myosteatosis is an accumulation of AC within skeletal muscle, known as intermuscular fat. There are several stem cell populations in skeletal muscle, the most well defined being muscle satellite cells (SCs), which lie below the basil lamina of muscle fibers and contribute to myogenesis during the process of muscle regeneration. A second, more recently described, population of cells is termed fibro/adipogenic progenitors (FAPs) or mesenchymal interstitial cells [Figure 1; Ref. (32–35)]. These cells are distinct from SCs and lack Pax7 expression but are Sca-1 and PDGFRα positive. SCs are generally resistant to adipogenic differentiation, whereas FAPs readily differentiate into ACs under various conditions such as muscle injury or glucocorticoid treatment (34, 36). Endogenous glucocorticoid levels increase with age (37), which may contribute not only to accumulation of bone marrow ACs but also to the deposition of intermuscular fat with age. Multipotent mesenchymal stem cells and other progenitors may also contribute toward skeletal muscle adipogenesis. For example, PW1^+^ interstitial cells (PICs) have shown adipogenic potential *in vitro* (38); however, the extent to which this population overlaps with FAPs is unclear. Additionally, type-1 pericytes expressing PDGFRα have been shown to commit to the adipogenic lineage *in vivo* in the presence of glycerol (39).

**Figure 2:**
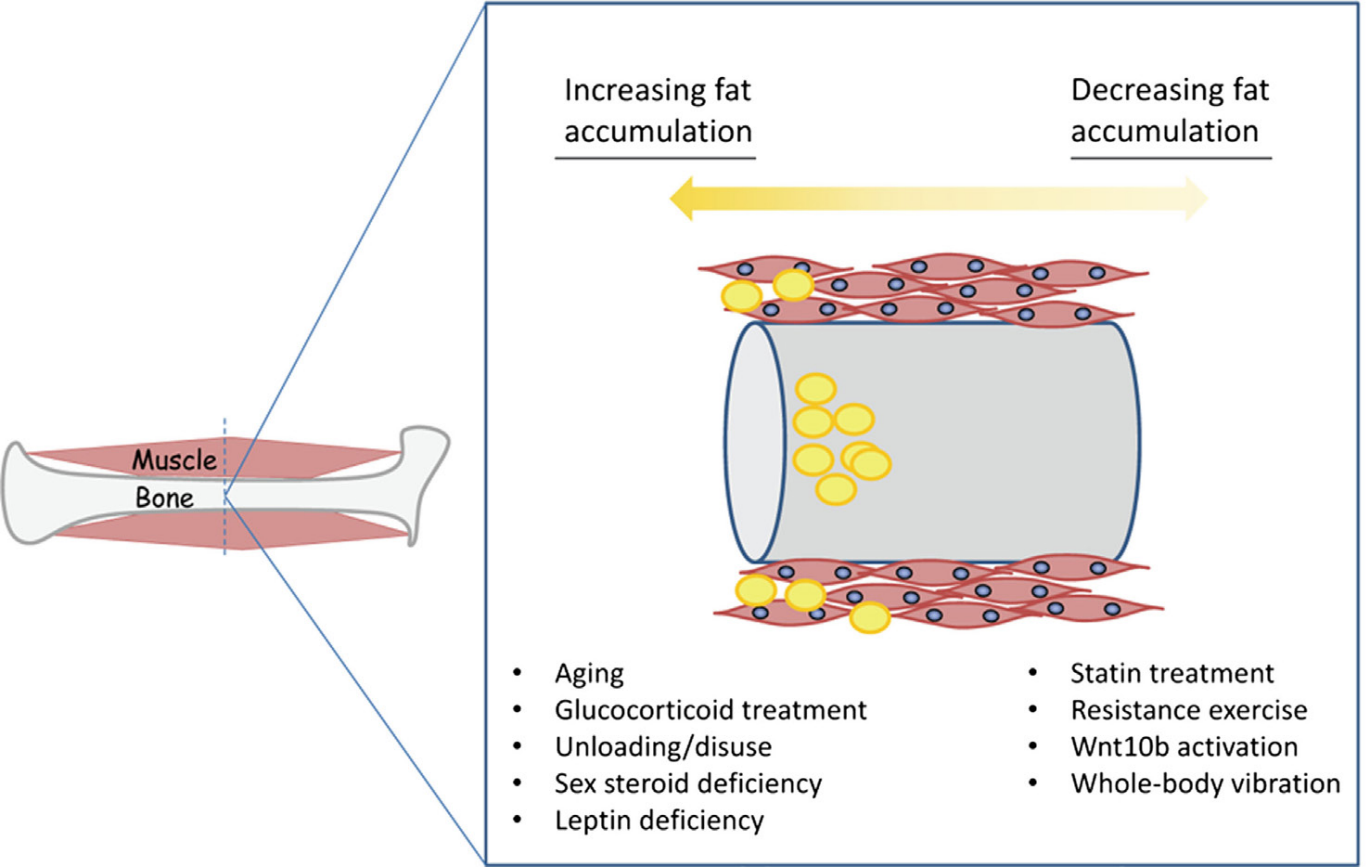
**Conditions favoring the accumulation of fat (yellow) in muscle and bone versus those conditions that can either prevent or possibly reverse fatty deposition in muscle and bone**.

**Figure 3:**
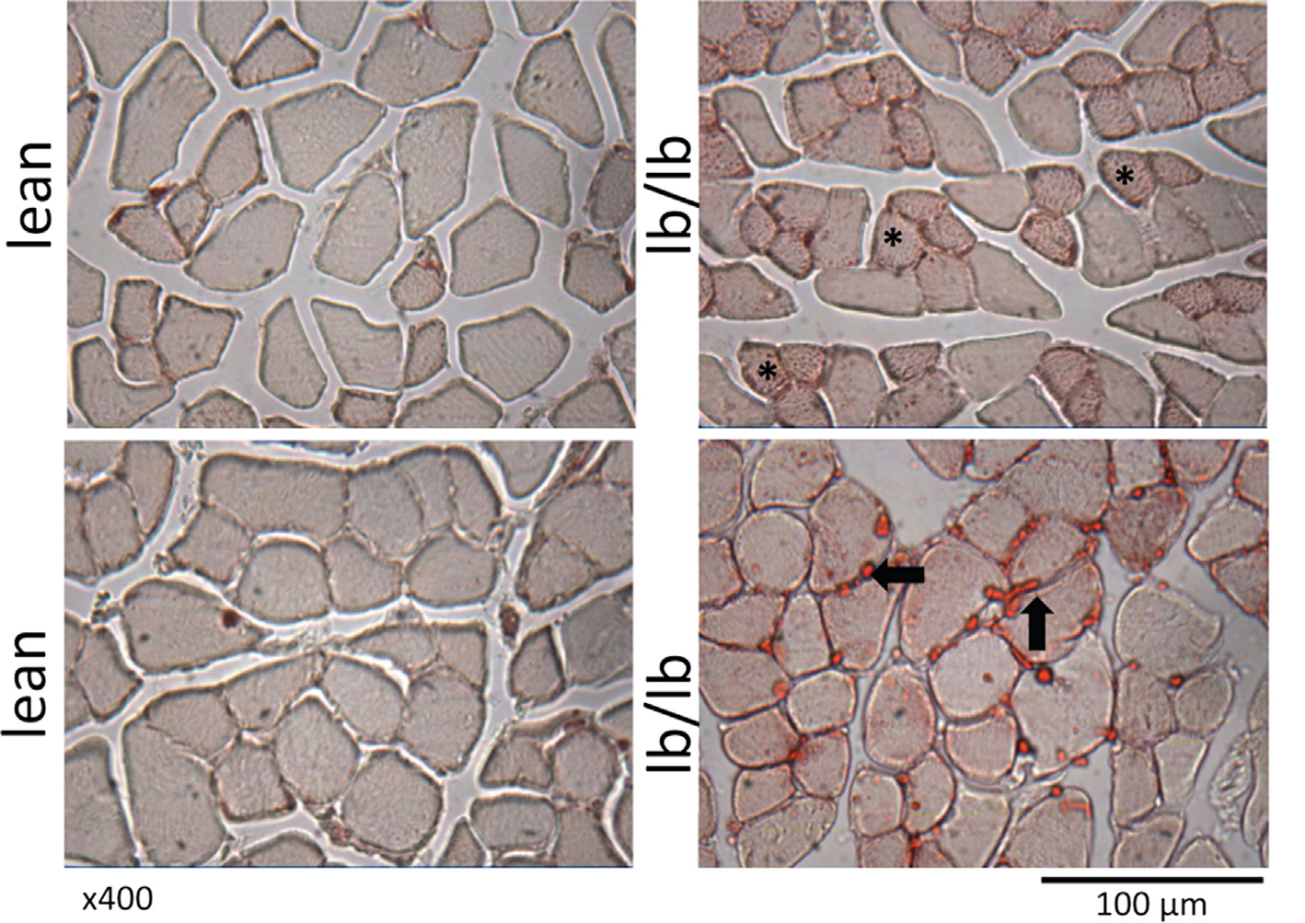
**Cross sections of muscle fibers from normal, lean mice (left), or mice that lack leptin receptors (lb/lb, right) stained for oil red O**. The asterisks indicate the accumulation of intramyocellular lipid, and the arrows indicate intermuscular fat. Note also the relatively small diameter of fibers that are positive for intramyocellular lipid. POUND mice (lb/lb) lack both short and long forms of the leptin receptor and are obese and hyperphagic (47).

Just as glucocorticoids can stimulate adipogenesis in both bone and muscle, other signaling pathways appear to be shared that regulate adipogenesis in muscle and bone (Figure 2). Wnt10b is well recognized to inhibit adipogenesis and stimulate bone formation in bone tissue (40). Wnt10b also suppresses the accumulation of IMC lipid in myofibers, increases insulin sensitivity, and inhibits adipogenic differentiation of aged, muscle-derived stem cells (41, 42). Similarly, inhibition of histone deacetylases (HDAC) can inhibit the adipogenic differentiation of MSCs *in vitro* and enhance their differentiation to osteoblasts (43), and HDAC inhibitors also inhibit the adipogenic differentiation of FAPs during the process of muscle regeneration (44). Altered leptin signaling, either due to absence of leptin or leptin receptors, is associated with increased bone marrow fat (45) as well as increased intra- and intermuscular fat (Figure 3). The leptin receptor is a key marker of bone marrow mesenchymal stem cells that mediate marrow adipogenesis (46), and the leptin receptor is also expressed in skeletal muscle (47). Whether or not the accumulation of inter- and intramuscular fat is directly mediated by the leptin receptor is, however, not well understood. Leptin deficiency associated with calorie restriction results in increased marrow adiposity (12), as does anorexia nervosa (48), but calorie restriction decreases lipid stores and lipid droplet size in skeletal muscle (49).

Unloading through either prolonged bedrest or spaceflight increases bone marrow adipogenesis (11, 14), and prolonged bedrest also decreases muscle strength and increases IMC lipid in skeletal muscle (50), which can ultimately lead to postural instability (51). Finally, estrogen deficiency is implicated in bone loss and marrow AC accumulation in women. Estrogen deficiency increases lipid content in skeletal muscle, the expression of adipogenic genes, and decreases relative satellite cell proportions in ovariectomized rodents (52, 53). Androgen deprivation therapy also increases fatty infiltration of skeletal muscle in men with prostate cancer, although CT imaging does not enable a distinction between IMC or intermuscular lipid accumulation and so the actual site of lipid deposition is not clear in this case (54). Together, these findings indicate that many of the conditions that induce marrow adipogenesis and bone loss in men and women such as disuse, sex steroid deficiency, altered leptin signaling, and glucocorticoid treatment also stimulate the accumulation of ACs and IMC lipid in skeletal muscle (Figure 2).

## Functional Consequences of Fatty Infiltration in Muscle

Protein synthesis enhances muscle hypertrophy and the maintenance of muscle strength, whereas impaired protein synthesis contributes to muscle atrophy. Insulin is an anabolic factor for skeletal muscle, and accumulation of muscle ACs and IMC lipid decreases insulin sensitivity, impairing the capacity for normal protein synthesis in skeletal muscle (30). Thus, decreased insulin sensitivity with fatty infiltration in skeletal muscle is one pathway by which fatty infiltration can directly affect muscle mass and muscle strength. The accumulation of IMC lipid with aging or with disuse is not homogenous across different muscles or different fiber types. This may be analogous to the unequal distribution of ACs throughout bone marrow in the appendicular skeleton, where fatty infiltration begins at more distal skeletal locations (55). For example, within the posterior compartment of the leg, the gastrocnemius accumulates more lipid with age than other calf muscles (21). Type I fibers, also referred to as “slow-twitch oxidative fibers,” tend to accumulate more IMC lipid with age in human subjects than fast-twitch oxidative fibers (23, 25), and fast-twitch fibers typically show greater atrophy with age than type I fibers (23, 56). It is possible that lipid accumulation alone may even support a transition of type II fibers to more of a type I phenotype (57). These changes ultimately lead to muscles with impaired contractile capacity of both type I and type II fibers, which together lead the dramatic decrease in muscle power (product of force and speed) observed with age (58).

Aging and disuse can induce the accumulation of IMC lipid, but muscle injury is associated with a marked accumulation of intermuscular fat (ACs), likely derived from the FAPs referenced above. This phenomenon has been most well described in patients with Duchene muscular dystrophy (DMD), where the prolonged cycle of muscle injury and regeneration that accompanies dystrophin deficiency ultimately results in an accumulation of ACs and fibrous tissue in areas where muscle fibers are lost (59, 60). The loss of muscle fibers and replacement with fatty and fibrous tissues leads to muscle weakness. The extent to which muscle injury with aging, which might occur with frequent eccentric muscle contractions, contributes to accumulation of intermuscular fat is not well documented. Fatty infiltration of skeletal muscle is also common following rotator cuff muscle injury and is a major factor that limits functional recovery (61). Attenuation of fatty infiltration following rotator cuff injury with statin treatment can have a protective effect on muscle atrophy in rats (62); however, a number of studies in human subjects indicate that fatty infiltration and muscle atrophy after rotator cuff repair is very difficult to reverse (63, 64). Hyperlipidemia and type 2 diabetes are independent risk factors for rotator cuff injury (65). It is certainly possible that these risk factors may not only increase the risk of rotator cuff injury but also may contribute to an attenuated repair response following treatment by exacerbating fatty infiltration of the injured rotator cuff muscles.

## Discussion: Targeting Adipogenesis and Lipid Accumulation in Muscle to Prevent Fracture

One of the most effective countermeasures against fatty infiltration of muscle with aging is physical activity and regular exercise. Previous work indicates that 6 months of regular aerobic exercise combined with weight loss reduced low-density muscle (lipid measurement) and improved glucose tolerance in men aged 60+ years compared with those who just exercised alone (66). Resistance training 3 days/week in adults’ age 55+ years decreased thigh intramuscular adipose tissue (67), and 1 year of brisk walking prevented fatty infiltration of muscle in older subjects (68). Importantly, resumption of physical activity following periods of sedentary activity could reverse the fatty infiltration that occurred in older adults following cessation of resistance training (69). Fracture risk in women declines with higher levels of weekly physical activity (70), and hip fracture in men is more common in those individuals with low physical activity compared with men with higher levels of physical activity (71). Resistance exercise increases leg strength and power in both older (aged 70 years) men and women (72), and this increase is associated with increased muscle fiber size (73). While the effects of exercise on bone and perhaps bone marrow ACs are more modest (74), resistance training may have a positive effect on reducing fracture risk by reducing intramuscular fat and increasing muscle strength and power.

Alternative forms of mechanical signals that are safe and can help prevent accumulation of muscular or bone marrow fat may be desirable, particularly, for the elderly or injured who are unable to exercise or have increased risk of fracture. Low magnitude (<1 *g*; *g* = earth’s gravitational field), whole body vibration has been observed to reduce adipose tissue as well as the expression of adipogenic genes in muscle (53, 75) while also acting as an anabolic signal and increasing muscle fiber area (76). Similarly, vibration has reduced bone marrow adiposity in a model of postmenopausal osteoporosis (77) and reduced bone marrow-derived mesenchymal stem cell commitment to the adipogenic lineage (78). Reduced indices of adipogenesis with the application of these mechanical signals as seen in both muscle and bone may occur through a similar mechanism – bias of mesenchymal stem cell or fate away from the fat differentiation pathway. These findings suggest that mechanical stimulation in a relatively low magnitude, high-frequency domain may have the potential to preserve muscle function with age by reducing the accumulation of lipids and ACs in skeletal muscle.

## Author Contributions

MH wrote the initial draft and prepared the manuscript illustrations. MM-L contributed additional narrative material on bone marrow adipogenesis and edited the manuscript. DF contributed narrative material on exercise and whole body vibration and on myosteatosis. DF also edited the manuscript.

## Conflict of Interest Statement

The authors declare that the research was conducted in the absence of any commercial or financial relationships that could be construed as a potential conflict of interest.
